# 6th International Conference on Typhoid Fever and Other Salmonelloses, Guilin, China, 12–14 November 2005

**Published:** 2007-03

**Authors:** A.D. Steele, B. Ivanoff

**Affiliations:** ^1^ Initiative for Vaccine Research, World Health Organization, Avenue Appia 20 CH-1211 Geneva 27, Switzerland; ^2^ International Vaccine Institute, Kwanak PO Box 14, Seoul 151-600, South Korea

The International Conference on Typhoid Fever and Other Salmonelloses is organized every two to three years and has usually taken place in Asia. The first meeting was held in Malaysia 15 years ago. The 6th international meeting, held in Guilin, China, in November 2005, was organized by the Guangxi Centre for Disease Prevention and Control, in collaboration with the World Health Organization (WHO), the International Vaccine Institute (IVI), and the American Society for Microbiology (ASM). Two hundred fifty participants from 27 countries, including Africa, participated.

Nine sessions covering the major topics of interest provided the latest updated information on typhoid fever. These include: Epidemiology and disease burden; Molecular pathogenesis and genomics; Contemporary issues in vaccine development; Diagnostics in typhoid fever; Antimicrobial resistance in typhoid fever; Clinical and therapeutic aspects; Socio-behavioural research; Other salmonelloses-related contemporary issues; and a Roundtable on Expanding Vaccine Implementation in Developing Countries.

Travel grants for participants from developing countries were supported by the WHO, IVI, and the ASM, and by the Conference Organizing Committee.

## Epidemiology and disease burden

During the epidemiology session, general consensus was raised that typhoid fever remains an important, but under-estimated, disease which has high mortality in developing countries, particularly in Asia. However, there are extremely limited data from Africa, and the burden of disease is probably under-estimated in this region. Endemic disease in South-East Asia is observed to be high, although it varies greatly in different settings. In addition, disease in the paediatric population was higher than previously recognized.

The latest published figure for the estimated number of cases is approximately 22 million per year, with an estimated 210,000 deaths (1). This number of deaths was calculated from a conservative figure using a case-fatality rate (CFR) of 1%. However, the reported CFR from presentations during the meeting was generally much higher than this in different studies, with the general agreement that this 1% estimate was under-estimated and that additional robust studies are required. It was suggested that WHO should convene a consultative meeting to resolve this during 2006. Second, the increasing number of *Salmonella* Paratyphi A isolates recovered in Asia, particularly in China and Pakistan, was noted with concern. In Guingxi province of China, 94% of *Salmonella* isolates are currently *S*. Paratyphi A. This situation raised the point of accelerating the development of *S*. Paratyphi A vaccine candidates.

## Molecular pathogenesis and genomics

Developments in molecular pathogenesis and genomics highlighted the difficulties that surround the serotyping of *Salmonella* species and the classification of *Salmonella* strains. The full genome sequence of *Salmonella* strains indicates that the *S*. Typhi and *S*. Paratyphi A have a recent evolutionary history. Using this sequence data, a novel system based on multi-locus sequences typing (MLST) has been devised which should help type *Salmonella* strains in a more precise approach and could replace classical serotyping in future. New data from Viet Nam showed that a haplotype of the TNF region was associated with fever-clearance time in patients. Individuals who have the protective haplotype (*12*22**1) cleared their fever significantly faster than individuals who did not have this haplotype. Better understanding of the organization of *Salmonella* pathogenicity islands and diversity of Typhi flagellar antigen expression were presented. Finally, it was recommended that further research was required on (a) *S*. Typhimurium in Africa, (b) paediatric versus adult strains of *S*. Typhi, and (c) molecular epidemiology of outbreaks versus endemic strains.

## Contemporary issues in vaccine development

Besides the currently-licensed and available vaccines (Vi polyssacharide and Ty21a), other candidate vaccines, consisting of either subunit vaccine or live-attenuated vaccines, were described. The Vi polysacharide vaccine is now locally produced in several developing countries, such as China, Viet Nam, and Cuba. This vaccine, extensively evaluated in Asia, through the DOMI programme of IVI, Seoul, demonstrated a confirmed protective efficacy of more than 70% when used in one injection, either in prevention in endemic areas or during an outbreak of typhoid fever. This sugar-based vaccine does not confer long-lasting immunogenicity in infants aged less than two years, and therefore, a conjugate vaccine was developed by National Institutes of Health (NIH), USA, and evaluated in young children. A phase III efficacy study in Vietnamese children, aged 2–5 years, demonstrated a protective efficacy of 91% after 27 months of active surveillance. Passive surveillance for another 16 months (3½ years of total follow-up) showed 82% protection against the infection. Evaluation of the Vi conjugate vaccine has also been conducted in infants aged 9–17 months. A live-attenuated oral dose candidate vaccine (Ty800), evaluated in in-patients, showed that a single, oral dose was well-tolerated and immunogenic over a wide dose range, and no subjects showed any signs of vaccine bacteraemia. Phase I/II studies are planned.

Another live-attenuated single oral dose candidate (CVD 909 developed by the Center for Vaccine Development, Baltimore), which produces both Vi and O antibody responses in a murine model, is under evaluation. In Mexico, a *S*. Typhi OMP (outer-membrane protein)-based approach was discussed showing that the OMPc produced a long-lasting immune response in mice >300 days. Induction of specific bactericidal antibody and cellular immune response was also reported in healthy volunteers. Because of the increasing number of *S*. Paratyphi A isolates, research is also focusing on the development of vaccine against infection due to this organism. Therefore, two approaches—live and conjugate vaccine development—were taken. A live-attenuated anti-typhoid/paratyphoid A and B single oral dose vaccine is under development in Switzerland. The vaccine strains were generated by a non-recombinant technology. A *S*. Paratyphi A conjugate vaccine, developed in China, in collaboration with NIH, was evaluated successfully in animals, and there are plans for its clinical evaluation. Food and Drug Administration (FDA)/Center for Biologics Evaluation and Research (CBER), USA, is using Ty21a as a live-attenuated vector for developing a multi-valent vaccine that has the potential to provide protection against typhoid fever and shigellosis.

## Diagnostics in typhoid fever

Discussions concerning the ongoing problems with diagnosis of typhoid fever and the development of new assays were raised. The clinical diagnosis of typhoid fever could be confused with other long-lasting fevers, and therefore, it was important to have a reliable and early diagnostic assay for this infection. Blood culture remains the definitive assay, but is known to miss cases by as much as 50%. The currently-available rapid tests (Typhidot and Tubex), evaluated in Asia, Pakistan, Bangladesh, and the Philippines, showed improved diagnostic reliability. New-generation tests, such as Dipstick-IgM and Typhi-rapid tests, have been developed for the early detection of IgM antibodies. These new-generation tests are very rapid taking from 10 to 15 minutes per test and for at least one manufacturer advances allow the testing of haemolyzed blood. The remaining issues included the great lot-to-lot variation with each of the available tests, which makes comparability from site to site or year to year very difficult. This issue is being improved by the manufacturers. A new rapid antibody-detection test for *S*. Paratyphi A-associated infection was presented. Even if these rapid tests can still be improved, they remain an alternative tool for the diagnosis of typhoid fever in the field.

## Antimicrobial resistance in typhoid fever

Antibiotic resistance remains an important problem in Asia and in Africa because of the possibility to buy antibiotics across the counter in the open market. Susceptibility patterns of *S*. Typhi strains showed high rates of multidrug resistance (MDR) in Asia and in Africa, with rates of 40% of isolates from India, 70% from Pakistan, 40% from Bangladesh, and 77% from Viet Nam. It was reported that more than 80% of isolates in Nigeria and 60% in Cameroon had resistance against ciprofloxacin and ceftriaxone. A three-year study in Iran showed a frequency of MDR of 50%, 67%, and 33% respectively in different Iranian locations. A non-plasmid-mediated fluoroquinolone-resistant *S*. Paratyphi A was reported from India. In addition, resistance of *S*. Typhi to nalidix acid varied between none in Laos to low levels in isolates from China (5%), higher rates on the India sub-continent (38%–Pakistan, 40%–Bangladesh, 61%–India), and higher levels in Viet Nam. Resistance of *S*. Paratyphi A to nalidixic acid was also reported in South China. Usually, the resistance to nalidixic acid is considered a marker for reduced susceptibility to fluoroquinolones. It is still not known if the NaR in *S*. Paratyphi A has a clinical profile similar to resistance of *S*. Typhi to nalidixic acid.

## General Discussion

Clinical presentations, including cardiopulmonary manifestations, ileal perforation, carditis, and hepatitis in relation to typhoid fever cases, were discussed. Socio-behavioural research outlined the importance of studies on the acceptability of vaccine and uptake for the management of vaccine campaigns during a trial and for interpretation of disease patterns during vaccine-efficacy evaluation.

During discussion in the final session, the issue of the target age for vaccination to prevent typhoid fever was raised. Based upon the epidemiological data, the optimal window to immunize would be from 2 to 20 years of age. Some studies showed that infants aged less than two years could be infected by *S*. Typhi, although they did not develop a real clinical typhoid fever. Moreover, neither vaccine is recommended as effective in infants aged less than two years, although the Ty21a vaccine was not evaluated in this age range. The Vi conjugate vaccine is expected to be immunogenic in such infants. Finally, reference was made to the recommendation of WHO on vaccination which should be conducted in large-scale nursery-based and school-based immunization programmes wherever the control of the disease is a priority with multidrug resistance strains of *S*. Typhi (2, 3).

In conclusion, there was a consensus to recognize that infection caused by *S*. Typhi still remains an important public-health problem, particularly in Asia and probably in Africa. The need was expressed to arrange a meeting to re-visit the currently-available data on the estimates of morbidity and mortality due to typhoid fever. There was a concern about the worldwide spread of multidrug resistance strains and the increasing emergence in China and Pakistan of *S*. Paratyphi A isolates. Finally, it was noted that, despite the fact that typhoid fever vaccines are shown to be effective, either in preventing or during an outbreak of typhoid fever, there is still some reluctance towards vaccination as a public-health intervention, probably due to lack of information. The future uptake and recommendation of the implementation of typhoid fever vaccines needs to be addressed by the international community and should be the topic of a future meeting by WHO.

## References

[1] Crump JA, Luby SP, Mintz ED (2004). The global burden of typhoid fever. Bull World Health Organ.

[2] World Health Organization (1998). Report of the Scientific Group of Experts (SAGE).

[3] Clemens CJ, Chandra-Mouli V, Byass P, Ferguson BJ (1999). Global strategies policies and practices for immunization of adolescents: a review.

